# Supplementary material for: Time-to-event estimation of birth prevalence trends: A method to enable investigating the etiology of childhood disorders including autism
Source: PLoS One. 2021 Dec 2;16(12):e0260738. doi: 10.1371/journal.pone.0260738 (PMC8638887; doi:10.1371/journal.pone.0260738)
Supplement: S1 Text — Contains figures. (PDF) [file pone.0260738.s001.pdf]

S1 text. TTEPE additional analyses and explanations.

Problems with age-period-cohort analysis

Rodgers [1] states about constraints added to enable identification “in fact [it] is exquisitely precise and has effects that are multiplied so that even a slight inconsistency between the constraint and reality, or small measurement errors, can have very large effects on estimates.” O’Brien [2], in a book devoted to this topic, states, regarding the relationships of age, period and cohort to the dependent variable, “There is no way to decide except by making an assumption about the relationship between these three variables.” MacInnis [3] showed that the effect of the set of diagnostic factors is represented by the years of first diagnoses, formulates the problem as one of separating birth year from diagnostic year, and shows that age-period-cohort approaches are not suitable for such analyses. In particular, implicit assumptions to make the model estimable cause the resulting estimates to conform to the assumptions, forming circular logic.

Ambiguity in estimation

To motivate the development of TTEPE, consider the ambiguity inherent in analyzing birth prevalence trends using cumulative incidence. How should one interpret a dataset that produces any one of the cumulative incidence curves illustrated in Fig 1? The figure represents synthetic data; some real-world data may be similar. Observed data might produce a curve resembling any one of the curves in the figure. An exponential curve with a coefficient of 0.1 fits all three plotted lines reasonably well. Does this represent a true increase in birth prevalence with a coefficient of 0.1? Does it result from an exponential increase in the effects of diagnostic factors — the probability of diagnosis — with no increase in birth prevalence? Perhaps a combination of both? The three similar cumulative incidence curves represent quite different possible explanations. The information shown in Fig 1 is not sufficient to decide which explanation most closely represents reality.

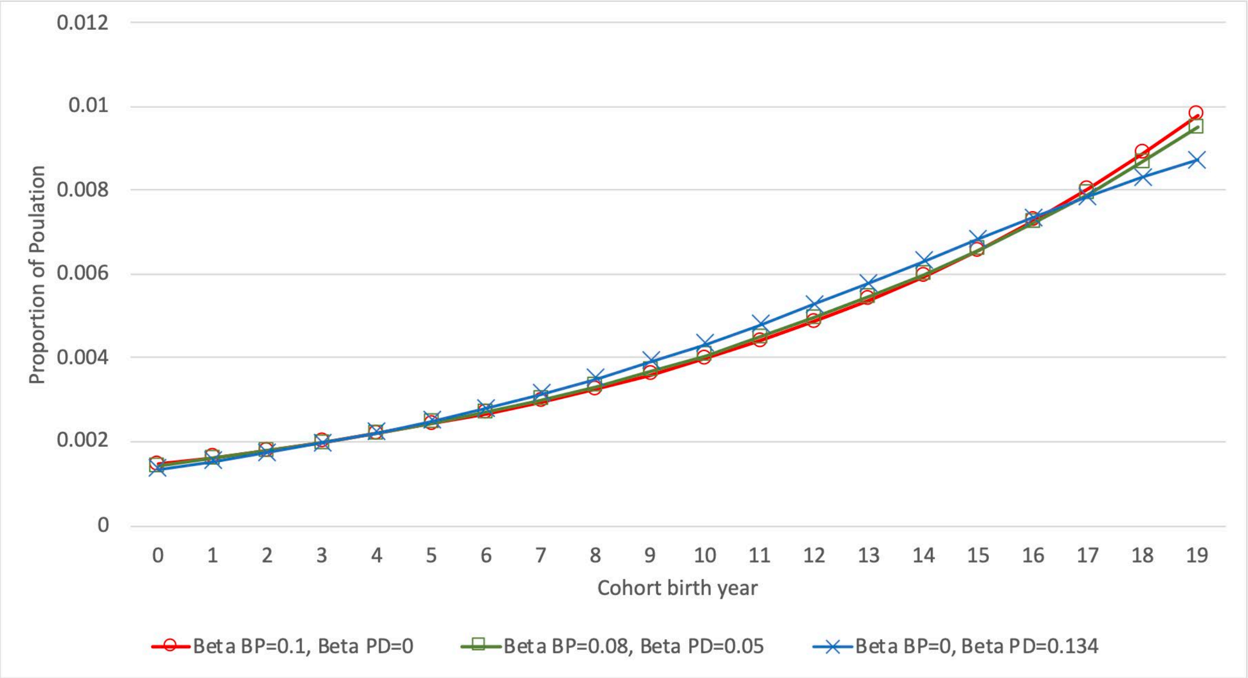

Fig 1. Example of cumulative incidence under three models. β_{BP} is the coefficient for birth prevalence; β_{PD} is the coefficient for the probability of diagnosis. Red line with circles represents $\beta_{BP} = 0.1$, $\beta_{PD} = 0$; green line with squares represents $\beta_{BP} = 0.08$, $\beta_{PD} = 0.05$; blue line with crosses represents $\beta_{BP} = 0$, $\beta_{PD} = 0.134$.

Fig 1 illustrates a hypothetical example of cumulative incidence of diagnoses to age ten over 20 consecutive cohorts using synthetic data. The legend lists the parameter sets for the three cases. β_{BP} is the exponential coefficient of birth prevalence BP by birth year and β_{PD} is the exponential coefficient of the probability of diagnosis PD by diagnostic year. In the case where $\beta_{BP} = 0.1$ and $\beta_{PD} = 0$, birth prevalence increases at $e^{0.1} - 1 =$ 10.5% per year while the probability of diagnosis is constant over time. Where $\beta_{BP} = 0$ and $\beta_{PD} = 0.134$, birth prevalence is constant while the probability of diagnosis increases by $e^{0.134} - 1 =$ 14.3% per year. Where $\beta_{BP} = 0.08$ and $\beta_{PD} = 0.05$, birth prevalence increases at 8.3% per year and the probability of diagnosis increases at 5.1% per year. The data generating process producing these data uses a survival process as detailed in the main paper. An Excel spreadsheet to generate all plots in this paper is available at OSF [4]. The variable PD represents the probability of diagnosis, which is the effect of diagnostic factors. The values and trends of cumulative incidence do not provide enough information to discern the relative contributions of the trends in birth prevalence and the probability of diagnosis. While the three cumulative incidence curves appear similar, the age distributions of diagnoses are strikingly different for different parameter sets, as Fig 2 illustrates.

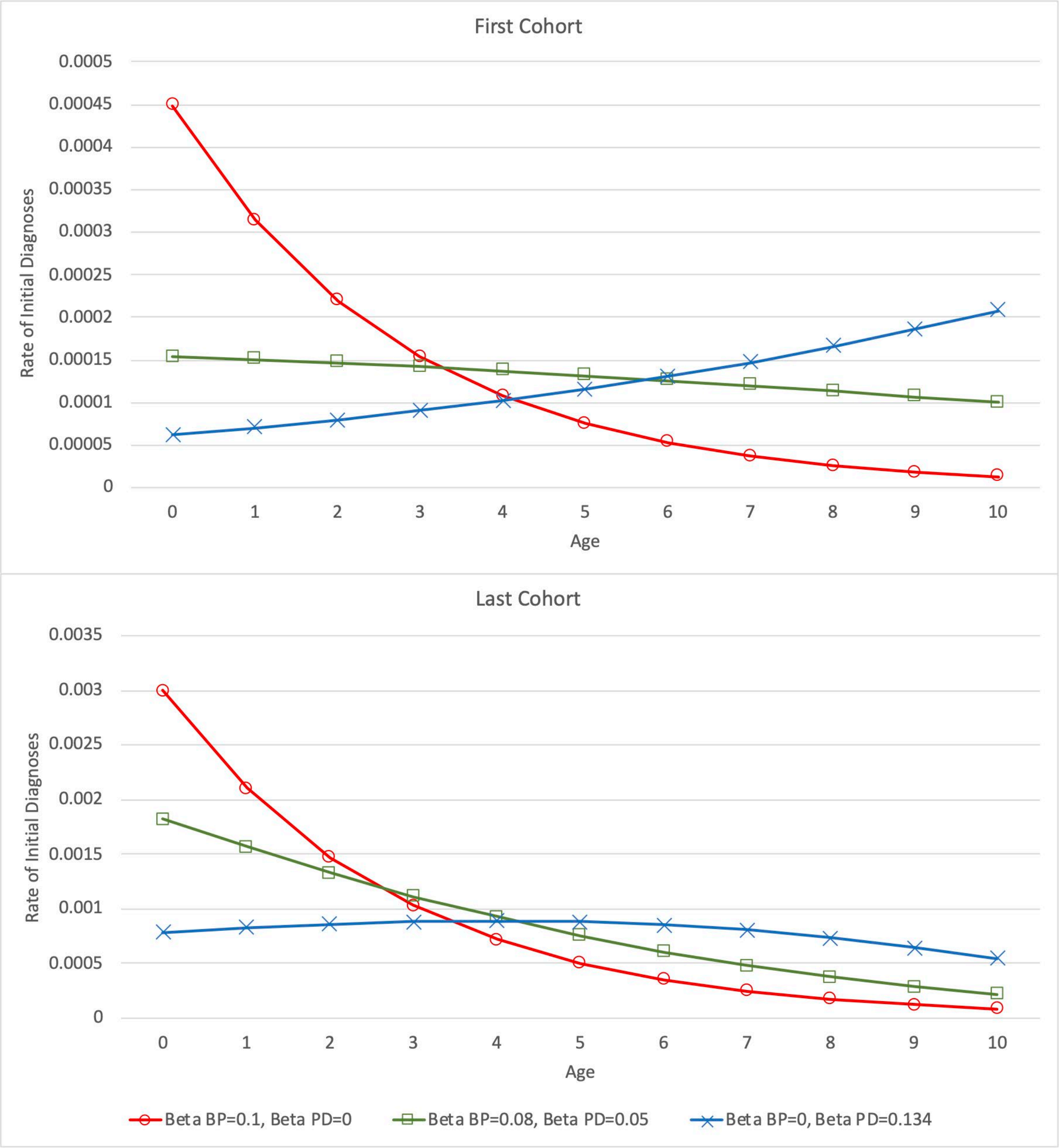

Fig 2. Distribution of diagnoses in the first and last cohorts under three models. β_{BP} is the coefficient for birth prevalence; β_{PD} is the coefficient for the probability of diagnosis. Red lines with circles represent $\beta_{BP} = 0.1$, $\beta_{PD} = 0$; green lines with squares represent $\beta_{BP} = 0.08$, $\beta_{PD} = 0.5$; blue lines with crosses represent $\beta_{BP} = 0$, $\beta_{PD} = 0.134$.

Fig 2 shows the age distributions of diagnoses in the first and last cohorts of Fig 1, with separate plot lines for each of the three parameter sets. For each cohort, the cumulative incidence to age 10 is very similar across all three parameter sets, despite the very different distributions of diagnoses by age between the parameter sets. The different values and trends in the

probability of diagnosis PD have a dramatic effect on the age distribution. The distinct age distributions associated with the different parameter sets are sufficient to ascertain the parameter values specifying the trends in Fig 2. The main paper explains how modeling the age distribution of first diagnoses enables accurate and unambiguous estimation of the coefficients for birth prevalence and diagnostic pressure.

References

1. Rodgers WL. Estimable Functions of Age, Period, and Cohort Effects. Am Sociol Rev. 1982;47(6):774-787.
2. O'Brien RM. Age-Period-Cohort Models. Boca Raton (FL): CRC Press; 2015.
3. MacInnis AG. Autism Prevalence Trends by Birth Year and Diagnostic Year: Indicators of Etiologic and Non-Etiologic Factors – an Age Period Cohort Problem [thesis]. Stanford (CA): Stanford University; 2017 DOI: 10.13140/RG.2.2.11821.59360. Available from: https://purl.stanford.edu/kz506jj7466
4. MacInnis AG. Time-to-event Prevalence Estimation TTEPE software and spreadsheet. DOI 10.17605/OSF.IO/WPNKU. Available from: https://osf.io/wpnku/
