# Supplementary material for: Time-to-event estimation of birth prevalence trends: A method to enable investigating the etiology of childhood disorders including autism
Source: PLoS One. 2021 Dec 2;16(12):e0260738. doi: 10.1371/journal.pone.0260738 (PMC8638887; doi:10.1371/journal.pone.0260738)
Supplement: Attachment — Submitted filename: Response to Reviewers.docx [file pone.0260738.s002.docx]

Dear Dr. Wang and the two reviewers of my PLOS ONE manuscript submission, PONE-D-20-24616, Time-to-event estimation of birth year prevalence trends: a method for investigating etiology of childhood disorders including autism.

Thank you very much for taking the time to review this submission and giving your comments. Your comments are very valuable to me. They provide insight into the ways that others may view the paper. Your comments have enabled me to improve the paper.

I have addressed all of your comments. Below is a copy of the body of the Decision email from February 16, 2021. I have interspersed my responses to your comments after each one, each one marked by “AGM:”

Reviewers' comments:

Reviewer's Responses to Questions

**Comments to the Author**

1. Is the manuscript technically sound, and do the data support the conclusions?

The manuscript must describe a technically sound piece of scientific research with data that supports the conclusions. Experiments must have been conducted rigorously, with appropriate controls, replication, and sample sizes. The conclusions must be drawn appropriately based on the data presented.

Reviewer #1: Partly

Reviewer #2: Yes

2. Has the statistical analysis been performed appropriately and rigorously?

Reviewer #1: I Don't Know

Reviewer #2: I Don't Know

3. Have the authors made all data underlying the findings in their manuscript fully available?

The [PLOS Data policy](http://www.plosone.org/static/policies.action#sharing) requires authors to make all data underlying the findings described in their manuscript fully available without restriction, with rare exception (please refer to the Data Availability Statement in the manuscript PDF file). The data should be provided as part of the manuscript or its supporting information, or deposited to a public repository. For example, in addition to summary statistics, the data points behind means, medians and variance measures should be available. If there are restrictions on publicly sharing data—e.g. participant privacy or use of data from a third party—those must be specified.

Reviewer #1: Yes

Reviewer #2: Yes

4. Is the manuscript presented in an intelligible fashion and written in standard English?

PLOS ONE does not copyedit accepted manuscripts, so the language in submitted articles must be clear, correct, and unambiguous. Any typographical or grammatical errors should be corrected at revision, so please note any specific errors here.

Reviewer #1: No

Reviewer #2: Yes

AGM: I revised the paper to make it clearer and easier to understand.

5. Review Comments to the Author

Please use the space provided to explain your answers to the questions above. You may also include additional comments for the author, including concerns about dual publication, research ethics, or publication ethics. (Please upload your review as an attachment if it exceeds 20,000 characters)

Reviewer #1: The paper addresses an important topic—i.e., changes over time in incidence and prevalence of chronic disorders, such as autism. By making us of full distributions of age-of-onset data, the proposed approach may well offer an important contribution.
Unfortunately, I find the paper as written to be difficult to understand. For example:
1. Many technical terms are used but only few are defined. As the author recognizes on p 3, technical terms, such as “incidence”, are often used in different ways. Thus, it is important to be clear about how all terms are used. Unfortunately, I often found definitions to be unclear. Examples include:
a. P3: “incidence” – “the rate of new cases” (p 3)—to me, this suggests that incidence should include reference to time, e.g., annual inference.

AGM: The revised version defines and clarifies incidence.

b. P3: “Prevalence” – “proportion of a defined population with the disorder at a defined time.” Does “time” here refer to year or age or something else?

AGM: The revised version defines and clarifies prevalence. The time of prevalence is the year when that prevalence applied; the study year. Prevalence, unless otherwise specified, is not specific to birth year or age.

c. P3: “birth year cohort prevalence”—birth year is not enough to define a population; one also needs to state the current age of that cohort

AGM: The revised version defines and clarifies birth year cohort prevalence and synonyms. Birth year prevalence is the set of people born in that year. It is an immutable construct that does not vary with age. Some portion of the cohort may have the disorder, and some portion of those show diagnosable symptoms at any given time.

Analysis can specify a cohort to comprise those who live in a geographic location at any given year. Individuals in the cohort may migrate into and out of the geographic location, so membership in the geographic specification of the cohort can change with time. Alternatively, analysis can specify a cohort as those born in a geographic location, even if they later migrate out.

d. P3: “disorder incidence” is “indistinguishable from birth prevalence”. I assume birth prevalence = birth year cohort prevalence. I think this can only be indistinguishable from disorder incidence if incidence is calculated over a time period equal to the current age of the cohort. But I’m not sure, because the use of terms is confusing me. For example, on p 5, the author states that CDC reports of 8 year-olds reflect “birth year prevalence.” This may be the same as the rate of new cases over the 1st 8 years of life, but I seldom see 8-year incidence reported. Note that some studies discuss “8 year probability” or “lifetime probability” of disorder to address this.

AGM: The revised version defines and clarifies these terms. The paper gives common synonyms for birth year prevalence, including birth year cohort prevalence, birth cohort prevalence, and birth prevalence.

The CDC ADDM reports are of the prevalence of autism or ASD for each birth year cohort. They determined prevalence using clinical evaluation of terms in the records for each subject and do not rely on cases having been diagnosed.

I infer that “8-year incidence” refers to the cumulative incidence of diagnoses by age 8. Some of the CDC ADDM papers talk about the proportion of the cases they determined to have ASD that were diagnosed by age 8; those diagnoses would form the cumulative incidence to age 8. Do “8 year probability” or “lifetime probability” mean the rate of diagnoses by age 8 or by end of life divided by the population? Or the probability that each case will be diagnosed by age 8 or by the end of life, i.e., conditional on being cases? The TTEPE paper addresses these topics using different words. The only way I know of to estimate the probability of cases being diagnosed by any particular age requires knowing how many cases there are, including those that have not yet been diagnosed. Please see the discussion of censoring in the paper, which explains how to estimate the proportion not yet diagnosed by the end of follow-up. One of the key variables in the paper is the probability of being diagnosed at each age, conditional on being an undiagnosed case, called diagnostic pressure, which is equivalent to hazard in survival or time-to-event analysis.

e. P3: “rate of incident diagnoses rather than true incidence”—again, I think I know what the author means by diagnoses vs “true” incidence, but I can’t be sure unless they are defined.

AGM: The revised version clarifies this. True incidence is the rate of occurrence of cases, whether or not they are diagnosed by any particular time or indeed ever. Incident diagnoses are new (first) diagnoses.

f. P 5: “cumulative incidence” is mentioned. How is this different than “birth year prevalence” assessed at a certain age?

AGM: The revised version clarifies this. Cumulative incidence of diagnosis is the accumulation of diagnoses by a specified age. Since not all cases are diagnosed by any particular age, and some may never be diagnosed, the cumulative incidence is generally less than the birth year prevalence. The difference between the two varies with birth year if changes in diagnostic factors explain part of an observed increase in the cumulative incidence of diagnoses over birth years.

g. P 20 “case prevalence” is mentioned. Do cases represent “true disorders” or only those who are “eligible” based on current symptoms?

AGM: The revised version clarifies this. Cases are those with the disorder whether or not they currently have diagnosable symptoms. Equivalently, cases are individuals for whom the disorder’s cause is complete, regardless of their symptoms. Eligible means that a case currently has diagnosable symptoms.

2. The paper is quite long and includes many examples that do not help to advance the argument.

AGM: I agree it is long. I wish I could make it shorter while explaining the subject matter and covering a broad range of contingent possibilities, but I am not sure that is possible. I added some material to clarify some potentially unclear points. I removed a few items.

For example:
a. there are numerous simulations, and I do not understand how Figure 1 helps

AGM: I added more explanation to the section “Ambiguity in estimation”, which contains Fig 1. I hope it is clearer now. This section shows why examining trends in cumulative incidence of diagnoses over time is insufficient to distinguish between trends in actual case prevalence and trends in diagnostic factors. Therefore we need a new method, which is what this paper presents.

b. The new method is not described in detail until p 16. There are many paragraphs with 1 or 2 sentences that seem to make an ancillary point (e.g., re: occum’s razor on p 14)

AGM: You are correct that there is substantial background material preceding the details of the method. My understanding is that this is necessary. If the paper were to jump quickly into the method, there would likely be many questions about it.

I removed the point about Occam’s razor. While it might be helpful for some readers, it is not necessary.

c. The DAG is not well-specified. For example, the author states that “diagnosable symptoms and diagnostic pressure together produce each initial diagnosis.” This sounds like an interaction to me, yet it is presented as if these were independent effects

AGM: The revised version clarifies the wording describing the DAG; among other things, it now says, “Diagnosable symptoms and diagnostic pressure interact to produce each initial diagnosis.” That is the same meaning as in the original version. I added the label “Diagnosable symptoms” to the figure. The text describes the DAG, and I do not see any remaining ambiguities in the DAG. Please let me know if anything about it is unclear.

d. The simulations are difficult to follow (at best), so I do not find them persuasive.

AGM: I clarified the wording regarding the simulation study. Please let me know if this is easier to follow. Simulation studies are fundamentally important for validating the behavior of statistical methods. The simulation study includes consideration of effects that could occur in real life or be suspected, and I believe it is important to show how much difference they can make. If you are interested in more information on simulation studies, I recommend Morris’s paper, cited in this paper.

At its core, the paper attempts to answer the question on p 4, “If reported birth prevalence increased over time, was this caused by changes in actual birth prevalence, the probability of diagnosing cases, diagnostic criteria affecting prevalence, or some combination of these factors?” As the author states later in the paper (p 7), many have discussed challenges to disentangling effects attributable to age, period, and cohort—i.e., quoting O’Brien who wrote, “there is no way to decide except by making an assumption about the relationship between these three variables.” In response, the author proposes a method based on several assumptions, yet notes often that it “correctly adjusts for changes in the set of diagnostic factors and diagnostic criteria.” The word “correctly” is used several times to describe the new method, but I don’t understand how this is appropriate when it is based on assumptions. For example, it is fine to assume that “diagnostic pressure” is equal across ages, but is this correct given that screening is only recommended at certain ages?

AGM: Thank you. I clarified the wording in the section on assumptions and some related points. As far as I know, all analysis methods have assumptions, even if they are not always clearly stated. Published papers that draw conclusions commonly do not address the validity of the assumptions that underlie the analysis. I made the list of assumptions as complete as possible to address any concerns readers might have that there might be unstated assumptions. The paper has a substantial discussion of possible violations of assumptions, how they might occur, and what investigators can do to detect them and address them. While this adds to the paper’s length, I feel it is appropriate to include all of that information.

I believe the method is correctly designed. It correctly resolves issues in existing methods, including age-period-cohort regression (which sometimes incorrectly omits one or more variables or assumes their values) and regression with improper adjustment for covariates. The paper discusses this. The paper shows how the TTEPE method is developed from first principles and is a minor extension to established time-to-event analysis methods. As far as I can tell, it is correct. The simulation study shows that the simulated models produce accurate estimates of the true parameters underlying the data. The paper discusses a broad range of things investigators can do to avoid introducing problems in analysis.

I removed several instances of “correctly” and added explanations clarifying the accuracy of the estimates.

I added a section that discusses the potential effect of screening on diagnostic pressure. It briefly describes how investigators can estimate that effect using TTEPE.

A more parsimonious argument would be helpful. As the author begins the discussion, “Readers may suspect that the estimates are unidentified, i.e., not unique, due to possible interaction between age, diagnostic year, and birth year, such that estimates may be biased even if the model fit is excellent.” It is not enough to state that “TTEPE avoids that problem and produces uniquely identified estimates.” As a reader who lacks the expertise to process the equations in detail (as I suspect many readers will be), a clear description is essential. For example, it is assumed that the eligibility function is consistent across cohorts (p 22) and that it is an “attribute of the disorder under study”—does this imply that the entire function is assumed, or just its functional form? How does TTEPE avoid identification problems when so many parameters (eligibility, incidence, diagnostic pressure, etc) are being estimated?

AGM: I would love to make the paper more parsimonious. Much of the substance of the paper is in response to experts’ comments from informal reviews. My understanding is that it is essential to include detailed explanations, discussion of a broad range of possibilities, the background, and simulations.

I revised the Model Fit and Discussion sections regarding identifiability and evidence of accurate estimates. The revised version points out that inappropriate models can fit the data well for certain datasets that coincidentally conform to implicit assumptions about the underlying parameters. This is a problem, for example, with age-period-cohort regressions.

I added some explanations to the equations to make them easier to understand. These include an explicit statement of the action of diagnostic pressure and the size of the risk set to produce the rate of diagnoses. I don’t think it would be appropriate to explain all of the equations in words, making the paper much longer. Of course, many readers will not be familiar with the math of time-to-event analysis, although some will be. Granted, it is tedious to go through it all carefully. I hope that the fact the equations use only basic algebra should help. The math is similar to that of the exponential distribution in survival analysis, with some additions.

I added some text in the Assumptions section regarding the consistency of the eligibility function. Eligible means having diagnosable symptoms, given that one is a case. The eligibility function gives the proportion of cases that are have diagnosable symptoms at each age. Eligibility being consistent across cohorts implies that the development of symptoms with age follows the same pattern in all of the cohorts.

I believe that relevant TTEPE models are identified because they avoid the sources of nonidentifiability in existing methods such as age-period-cohort, and TTEPE utilizes and fits the age distribution of initial diagnoses at all cohorts. The simulation study exercised the method and model over a full range of plausible parameter values and shows that it accurately estimates the true parameters across the range. I searched at length for TTEPE models that might be unidentified and did not find practical examples. I did find some impractical examples and alluded to them in the paper. For example, if one were to add an unconstrained non-parametric term for diagnostic pressure varying with age while analyzing only a single birth cohort, that can create a collinearity and the model can become unidentified. However, estimating all parameters simultaneously over a range of birth cohorts avoids that problem.

In the example models considered and simulated in the paper, there are 20 cohorts and 11 ages resulting in 220 data points. In these models, prevalence and diagnostic pressure are specified with two parameters each: the intercept (or final value) and the exponential coefficient. There are various ways one can specify the form of eligibility to enable estimation. The most unrestricted form would have 10 values, for ages 0 through 9, and setting the value at age 10 equal to 1, which is necessary for identifiability, as the paper says. That would be a total of 14 values to estimate from 220 data points, which should not be a problem. There are more parsimonious forms of eligibility, such as an exponential rise from age 0 to an age of eligibility (AE) above which eligibility E = 1, with three values to estimate (intercept, exponent and AE). Or, a form that supports an ongoing increase in eligibility with age, with 2 or 3 more values. A simpler approach is taken in the simulation study: Assume eligibility plateaus as some age, where the age can be based on external knowledge of the disorder or a preliminary estimation. In an extreme case, if prevalence, diagnostic pressure and eligibility were all specified non-parametrically, there would be 20 values for prevalence, 11 values for diagnostic pressure and 10 values for eligibility, for a total of 41 values. With the same 220 data values, the model standard error would be greater than what the simulation study found, but that would not make the model unidentified. Such an approach might be more suitable for exploring the data than for generating final estimates. Understanding the data based on such exploration can facilitate parsimonious specifications for each variable, leading to accurate estimates.

Clear and cogent answers to questions such as these will facilitate review of the merits of the proposed method and—assuming it is indeed correct—promote is wider dissemination.

AGM: Thank you.

Reviewer #2: This paper addresses a very important topic – monitoring the prevalence of ASD over time given changes in diagnostic practices. The emphasis, by virtue of including parameters of rates of initial diagnosis by diagnostic year and age for each birth cohort and using simulation studies to understand the benefits and limitations of the method is a novel and useful approach. Prior to presenting my review, I wish to disclose that while I have written papers on the prevalence of childhood disorders, have used survival analyses to model prevalence, and have been very interested in following ASD prevalence papers, I do not have the mathematical knowledge to evaluate this element of the modeling. This said, I believe that the author has done a great job of reviewing the challenges and controversies in this area, making a strong case for the need for new methods to disentangle potential changes in prevalence of ASD over time from diagnostic practice factors such as changing diagnostic criteria and greater outreach with screening. The presentation of the method is extremely clear.

AGM: Thank you.

This approach builds on survival analysis models in recognizing that risk for diagnosis continues through the lifespan as well as incorporating changing clinical practices in relation to diagnosing ASD and IDD.

AGM: Agreed, and thank you for this concise summary.

Distinguishing incidence and prevalence may be more important when conditions are not enduring or are episodic such as depression.

AGM: I am not sure of the significance of this point. Consider, for example, infectious diseases: incidence is of primary importance for understanding how to control the disease, while prevalence gives insights into the caseload of caring for individuals with the disease and predicting total mortality rates. For any disorder or disease, incidence is important for understanding its causes. For disorders that are present or predetermined at birth, the concept of incidence doesn’t have much of a practical interpretation because the disorder occurrence (or complete cause) is prenatal, and birth (year) prevalence is the practical epidemiologic metric.

I appreciate that the author highlights the potential for lack of homogeneity in diagnostic pressure within cohorts. Unfortunately, this is likely to be the case as regional variation in practice continues to be quite variable and health disparities may also contribute to heterogeneity in practice within cohorts.

AGM: Thank you for asking about this. The revised version has more specific language regarding elucidating the effects of regional variation and health disparities where data is available for analysis.

It might also be useful to recognize that ASD diagnoses generally begin to be possible in the second year of life with many cases emerging in the third year of life (see Zwaigenbaum et al., Ozonoff et al., who each follow diagnoses within infant sibling samples) and that there is greater instability in early diagnoses (i.e., under age 2) than when diagnoses are made in later preschool (e.g., Giserman Kiss & Carter, 2019).

AGM: Thank you. Yes, cases tend to start developing symptoms over approximately the first three years of life, and that is what the eligibility term in TTEPE represents. I added this suggested material to the revised manuscript, and discussed what effect it might have. Regarding cases diagnosed at early ages whose symptoms resolve later such that they would no longer qualify for the diagnosis, it is not clear what one would want to say about that. Those cases were cases, and TTEPE counts them as such, as I think it should. For cases diagnosed early whose symptoms progress with age they are likewise counted as cases in TTEPE. The papers you cited provide interesting insights into the progression of case symptoms with age. It would be interesting to investigate why that happens; that is outside of the scope of TTEPE.

The clarity of the writing is a major strength of this paper. My only suggestion is to incorporate more findings that impact the timing and heterogeneity of diagnoses into the discussion.

AGM: Thank you. The revised version has more discussion of this topic.

6. PLOS authors have the option to publish the peer review history of their article ([what does this mean?](https://journals.plos.org/plosone/s/editorial-and-peer-review-process#loc-peer-review-history)). If published, this will include your full peer review and any attached files.


If you choose “no”, your identity will remain anonymous but your review may still be made public.


**Do you want your identity to be public for this peer review?** For information about this choice, including consent withdrawal, please see our [Privacy Policy](https://www.plos.org/privacy-policy).

Reviewer #1: No

Reviewer #2: No

[NOTE: If reviewer comments were submitted as an attachment file, they will be attached to this email and accessible via the submission site. Please log into your account, locate the manuscript record, and check for the action link "View Attachments". If this link does not appear, there are no attachment files.]

While revising your submission, please upload your figure files to the Preflight Analysis and Conversion Engine (PACE) digital diagnostic tool, <https://pacev2.apexcovantage.com/>. PACE helps ensure that figures meet PLOS requirements. To use PACE, you must first register as a user. Registration is free. Then, login and navigate to the UPLOAD tab, where you will find detailed instructions on how to use the tool. If you encounter any issues or have any questions when using PACE, please email PLOS at [figures@plos.org](mailto:figures@plos.org). Please note that Supporting Information files do not need this step.

*In compliance with data protection regulations, you may request that we remove your personal registration details at any time.* [*(Remove my information/details)*](https://www.editorialmanager.com/pone/login.asp?a=r)*. Please contact the publication office if you have any questions.*
