# Supplementary material for: Time-to-event estimation of birth prevalence trends: A method to enable investigating the etiology of childhood disorders including autism
Source: PLoS One. 2021 Dec 2;16(12):e0260738. doi: 10.1371/journal.pone.0260738 (PMC8638887; doi:10.1371/journal.pone.0260738)
Supplement: Attachment — Submitted filename: Response to reviewers 29Sep21.docx [file pone.0260738.s003.docx]

Response to reviewers PLOS ONE PONE-D-20-24616R1

Review Comments to the Author

Please use the space provided to explain your answers to the questions above. You may also include additional comments for the author, including concerns about dual publication, research ethics, or publication ethics. (Please upload your review as an attachment if it exceeds 20,000 characters)

Reviewer #3: (No Response)

Reviewer #4: The paper addresses an important topic—birth year prevalence estimation adjusted for time-varying diagnostic factors and diagnostic criteria. By making use of the time-to-event survival method to model the causal paths from birth year, diagnostic year, and age at diagnosis, the proposed approach may well offer an important contribution. Unfortunately, even after the revision, this paper is still too long and difficult for the reader to understand.

Author’s response:

Thank you.

I restructured the paper to make it much easier to understand. One key part of that is the new section “Description of the method,” which is intended for a general audience. In addition, I moved substantial parts of the paper to supplemental material [S1 text], and removed other parts. I worked with a professional editor, who is not technical in this field, to ensure the paper is easy to understand and has maximum clarity. Moving and removing material reduced the length substantially while adding the description added back much of the reduced length. I believe it is worthwhile to have the new description section and keep all of the sections that remain in this revision.

The editor and I went over this several times, looking for opportunities to make it shorter without removing important information.

Please note that some of the material was specifically requested by a previous reviewer and I do not believe I am empowered to remove it.

For example:

1. There is a logical confusion between the description of “incidence” and “prevalence” in Introduction part. As the author states on p3, “Incidence is different from prevalence” and “Prevalence is rarely of direct interest in studying etiology”, does it seem unreasonable to use birth-year cohort prevalence rather than incidence in this paper? The definition and distinction between “incidence” and “prevalence” and why "prevalence" was chosen still needs further elaboration and clarification.

Author’s response:

I heavily revised the introduction to make all of this clear. It explains why birth prevalence, not incidence, is the appropriate measure to quantify the rate of occurrence of new cases of disorders and diseases where the occurrence of new cases is not observable because case onset is either before or shortly after birth, including autism. Birth prevalence is the proportion of a birth year cohort that has the disorder, regardless of when or whether each case was diagnosed. The occurrence of new diagnoses is observable, but those diagnoses may come much later than case onset, and some cases may never be diagnosed. The literature on autism, for example, frequently uses “incidence” to mean incident diagnoses rather than incident cases, often without clearly stating the meaning in use. The difference between the incidence of cases and incidence of diagnoses is fundamental to resolving the question raised by the hypothesis that observed increases in diagnosis result from increases in diagnostic factors rather than increasing case incidence or birth prevalence. The standalone term “prevalence” is different from both incidence and birth prevalence, regardless of whether incidence refers to new cases or new diagnoses. “Prevalence” is a measure of the total number of cases at a specific time, not the occurrence rate; it is not specific to birth year and is specific to time. The revised introduction explains all of that.

The revised introduction has definitions of all of the relevant terms, including birth prevalence, incidence, prevalence, cumulative incidence, diagnostic factors, probability of diagnosis and diagnostic factors. The revised paper consistently uses “birth prevalence” throughout, including the variable name *BP*. In addition, it states that birth prevalence has the synonyms “birth year prevalence” and “birth year cohort prevalence.” “Birth prevalence” is the more commonly used term in the literature. The previous version of this paper used multiple different terms to mean birth prevalence; I corrected that.

2. The article is too long and some parts do not need to be separate paragraphs. The Literature Review section is overly long and not very meaningful to the model presented in this article. The description of the strengths and weaknesses of the available methods needs to be further streamlined and summarized rather than simply listed. The Overview section needs to be further refined and integrated into the Introduction section.

Author’s response:

I removed a significant amount of material that was arguably either unnecessary or redundant. I moved another significant amount of material to a new supplementary file [S1 text]. And, I heavily revised the literature review to focus it directly on the question at hand, shorten it, and make it easier to understand. The literature review shows that numerous papers speculate that observed trends in birth prevalence, which they sometimes incorrectly label “prevalence,” are caused by diagnostic factors rather than actual birth prevalence, but without giving appropriate evidence and analysis, and none suggest methods to resolve the discrepancy properly. That background shows the need for a new method.

I merged the appropriate parts of the Overview into the Introduction. In addition, I either eliminated or merged short paragraphs as appropriate.

3. The Background part in Method of time-to-event prevalence estimation (TTEPE) appears to be a duplicate of Introduction and Literature Review.

Author’s response:

I eliminated all duplicative material. There is now one section, “Background and Literature Review.”

4. Ambiguity in estimation part does not seem necessary, and a more parsimonious argument would be helpful. A quick entry into the model specification would have been better for the reader to understand the point of the article.

Author’s response:

I moved the “Ambiguity in estimation” section to the new supplementary file [S1 text]. The revised paper goes directly into the description of the method immediately after the revised background and literature review. The description of the method is in plain English without math, directed towards maximum understanding by a broad audience. The following sections give the method details and the analysis model, including the derivation of formulas.

This paper uses a time-varying survival analysis model to portray birth year prevalence trend is reasonable and novel. While, the structure of the article still needs to be reorganized, and the key points needs to be sharpened rather than simply narrated.

Author’s response:

Thank you. I strongly agree that the TTEPE method is reasonable and novel. I restructured and heavily revised the paper according to your suggestions.

Changes to reference list:

All references in the March 2021 version are included in this new revision. The following references are additions in this version compared to the March 2021 version.

12.	Myers SM, Voigt RG, Colligan RC, Weaver AL, Storlie CB, Stoeckel RE et al. Autism Spectrum Disorder: Incidence and Time Trends Over Two Decades in a Population-Based Birth Cohort. J Autism Dev Disord 2019;49:1455–1474. https://doi.org/10.1007/s10803-018-3834-0

Meyers et al reported trends in cumulative incidence, approximating birth prevalence, and suggested that it might be the result of increased diagnosis and they have a dataset that may be suitable for TTEPE analysis.

16.	Sasayama D, Kuge A, Toiban Y, Honda H. Tends in Autism Spectrum Disorder Diagnoses in Japan, 2009-2019. JAMA Network Open. 2021;4(5):e219234. doi:10.1001/jamanetworkopen.2021.9234

Sasayama et al reported trends in cumulative incidence, approximating birth prevalence and suggested that it might be the result of increased diagnosis, and they have a dataset that may be suitable for TTEPE analysis.

19.	Hansen SN, Schendel DE, Parner ET. Explaining the Increase in the Prevalence of Autism Spectrum Disorders: The Proportion Attributable to Changes in Reporting Practices. JAMA Pediatr. 2015;169(1):56–62. doi:10.1001/jamapediatrics.2014.1893

Hansen et al reported a strong increase in birth prevalence and suggested that it might be the result of increased diagnosis, and they have a dataset that may be suitable for TTEPE analysis.

20.	Newschaffer CJ, Croen LA, Daniels J, Giarelli E, Grether JK, Levy LA, et al. The Epidemiology of Autism Spectrum Disorders. Annu Rev Public Health. 2007;28:235-258

Newschaffer et al wrote that it is “virtually impossible” to solve the problem of separating the effects of birth prevalence from diagnostic factors.

22.	National Birth Defects Prevention Network. Guidelines for Conducting Birth Defects Surveillance: Chapter 8, Statistical Methods. 2004. Available at: https://www.nbdpn.org/docs/Ch_8_Statistics6-04_2016DEC14.pdf

This NBDPN paper shows that “birth prevalence” is the appropriate statistical measure for birth defects.

23.	Centers for Disease Control. Birth Defects Surveillance Toolkit: 3.2 Population Coverage. Available at: https://www.cdc.gov/ncbddd/birthdefects/surveillancemanual/facilitators-guide/module-3/mod3-2.html. Accessed August 10, 2021.

This CDC instructional module shows that “birth prevalence” is the appropriate statistical measure for congenital anomalies.

24.	Larsson HJ, Eaton WW, Madsen KM, Vestergaard M, Olesen AV, Agerbo E, et al. Risk Factors for Autism: Perinatal Factors, Parental Psychiatric History, and Socioeconomic Status. Am J Epidemiol. 2005;161:916–925

Larsson et al give evidence that autism case status is determined by the time of birth and tends to depend on prenatal risk factors.

25.	Wolff JJ, Piven J. Predicting Autism in Infancy. J Am Acad Child Adolesc Psychiatry. 2021;60(8):958-967

Wolff and Piven show that autism is generally predetermined from birth and symptoms appear later.

28.	Sheldrick RD, Maye MP, Carter AS. Age at First Identification of Autism Spectrum Disorder: An Analysis of Two US Surveys. J Am Acad Child Adolesc Psychiatry. 2017;65(4):313:320

Sheldrick et al examine age distribution of initial diagnoses of autism and use survival analysis to estimate cumulative incidence. However, they use the entire cohort population as the denominator in their hazard estimate, similar to two Hansen et al papers. They point out that symptoms appear over time and may not be observable at young ages, and state that age of identification is related to symptom severity.

29.	American Psychiatric Association. DSM History. Accessed August 3, 2021. Available from: https://www.psychiatry.org/psychiatrists/practice/dsm/history-of-the-dsm

This APA report describes the history of DSM diagnostic criteria. Citing this is much shorter than summarizing the history in the present paper.

30.	Morris-Rosendahl DJ, Crocq MA. Neurodevelopmental disorders—the history and future of a diagnostic concept. Dialogues Clin Neurosci. 2020;22(1):65-72. doi:10.31887/DCNS.2020.22.1/macrocq

Morriss-Rosendahl and Crocq describe the history of both CDM and ICD diagnostic criteria.

40.	Hertz-Picciotto I, Delwiche L. The Rise in Autism and the Role of Age at Diagnosis. Epidemiology. 2009;20(1):84-90.

Hertz-Picciotto and Delwiche show that datasets exist for the California Department of Developmental Services that may be suitable for TTEPE analysis.

42.	Shattuck PT, Durkin M, Maenner M, Newschaffer C, Mandell DS, Wiggins L, et al. Timing of Identification Among Children With an Autism Spectrum Disorder: Findings From a Population-Based Surveillance Study. J Am Acad Child Adolesc Psychiatry. 2009;48(45):474-483.

Shattuck et al used survival analysis methods to estimate the median age at first diagnosis of autism.

43.	Maenner MJ, Schieve LA, Rice CE, Cuniff C, Giarelli E, Kirby RS, et al. Frequency and Pattern of Documented Diagnostic Features and the Age of Autism Identification. J Am Acad Child Adol Psychiatry. 2013;52(4):401-413

Maenner at al used survival analysis methods to estimate differences in survival curves between cases with different numbers of autism symptoms and different symptom severity.

48.	Lord C, Bishop SL. “Let’s Be Clear That “Autism Spectrum Disorder Symptoms” Are Not Always Related to Autism Spectrum Disorder”. Am J Psychiatry. 2021;178(8).

Lord and Bishop state that symptoms that may appear to be autism symptoms do not necessarily indicate the presence of autism.

Additionally, the following reference is unchanged except the revised version has a different, more concise URL to access the paper.

27.	MacInnis AG. Autism Prevalence Trends by Birth Year and Diagnostic Year: Indicators of Etiologic and Non-Etiologic Factors – an Age Period Cohort Problem [thesis]. Stanford (CA): Stanford University; 2017 DOI: 10.13140/RG.2.2.11821.59360. Available from: https://purl.stanford.edu/kz506jj7466
